# Pediatric patients with tenosynovial giant cell tumor: real-world evidence from an observational registry

**DOI:** 10.1186/s13023-026-04231-7

**Published:** 2026-01-30

**Authors:** Sydney Stern, Patrick F. McKenzie, Giacomo G. Baldi, Thomas J. Scharschmidt, Emanuela Palmerini, Sara Rothschild

**Affiliations:** 1 TGCT Support/Life Raft Group, 155 US Highway 46, Suite 202, Wayne, NJ 07470 USA; 2 Department of Organismic and Evolutionary Biology, Harvard University, Cambridge, MA USA; 3 Department of Oncology, Hospital of Prato, Azienda USL Toscana Centro, Prato, Italy; 4 Department of Orthopaedic Surgery, Nationwide Children’s Hospital, Columbus, OH USA; 5 Sylvester Comprehensive Cancer Center, Miller School of Medicine, University of Miami, Miami, FL USA

**Keywords:** Tenosynovial giant cell tumor, Rare tumor, Mesenchymal tumor, Observational registry, Oncology, Pediatrics, Comparative analysis

## Abstract

**Background:**

Tenosynovial giant cell tumor (TGCT) is a rare, locally aggressive tumor originating in the synovial lining of the joint, bursa, and tendon sheath. TGCT typically affects individuals between 20 and 50 years of age and pediatric cases are considered ultra-rare. Research and clinical trials thus far have been largely focused on the adult TGCT population. Therefore, data are needed to understand the impact of TGCT on pediatric patients’ quality of life and any differences between adults. Here we report the result of the pediatric TGCT population enrolled in an observational patient registry.

**Patients and methods:**

A total of 122 pediatric patients (9.5%) were included in this exploratory, cross-sectional analysis of a longitudinal 1,278-patient registry from October 06, 2022 to November 26, 2024. Among pediatric patients, 73.0% had diffuse TGCT (D-TGCT; *n* = 89), 16.4% had localized TGCT (L-TGCT; *n* = 20), and 10.6% had unspecified TGCT (*n* = 13). Pediatric patients had a median age at diagnosis of 14.5 years.

**Results:**

More than half of pediatric patients were initially misdiagnosed and were more likely to be misdiagnosed than adults (62.3% vs. 49.9%, *p* < 0.01) and received joint aspirations significantly more frequently than adult patients (47.5% [*n* = 58] vs. 22.5% [*n* = 112], *p* < 0.05). Most pediatric patients were diagnosed by orthopedic surgeons (*n* = 79, 64.8%), and 52.5% of pediatric patients were diagnosed ≥ 1 years after symptom onset. Pediatric patients with D-TGCT underwent an average of 3.4 surgeries, compared to 1.8 surgeries for those with L-TGCT. Recurrence rates were similar among adults and pediatric patients with 66.3% of pediatric patients with D-TGCT having ≥ 1 post-operative recurrence compared to 15.0% of L-TGCT pediatric patients, respectively. Despite no approved systemic therapies for pediatric use, pediatric and adult patients consulted medical oncologists in similar rates and systemic therapies were prescribed similarly but infrequently overall (*n* = 21, 17.2% in pediatric patients and *n* = 95, 19.1% in adults).

**Conclusions:**

Pediatric patients had significant disease burden, as compared to adults with TGCT, which severely affected their quality of life. The reliance on surgical treatment and underuse of multidisciplinary care emphasizes the unmet need for provider education and treatment advancements tailored to this population.

**Trial registration:**

This study was an analysis of an observational patient registry and therefore was not registered as a clinical trial; no trial registration number is available. The study protocol was approved by Advarra (protocol reference number: Pro00077310). Patient enrollment for this analysis occurred from October 6, 2022, to November 26, 2024.

**Supplementary Information:**

The online version contains supplementary material available at 10.1186/s13023-026-04231-7.

## Introduction

Tenosynovial giant cell tumor (TGCT) is a rare, locally aggressive, monoarticular, mesenchymal tumor affecting the synovium, bursa, or tendon sheath [1]. Historically, TGCT has been called pigmented villonodular synovitis (PVNS) and giant cell tumor of the tendon sheath. In 2013, the World Health Organization consolidated the nomenclature to define a single neoplasm known as TGCT with two distinct radiologic presentations: diffuse and localized subtypes [2]. Diffuse TGCT (D-TGCT) describes an extensive infiltrative involvement of the joint and may extend beyond the joint capsule into extra-articular structures, while localized TGCT (L-TGCT) describes a nodular and well-circumscribed lesion(s). TGCT may occur in any joint, but L-TGCT commonly affects the digits while D-TGCT instead commonly affects larger joints such as the knee, hip, and ankle [2, 3]. The various nomenclature used to describe the same condition has led to confusion among patients and providers as well as fragmented research. Importantly, TGCT now describes nodular tenosynovitis, giant cell tumor of the tendon sheath, PVNS, TGCT, and diffuse-type PVNS.

TGCT primarily affects adult patients between 20 and 50 years of age, with a median age in the 40s [4]. Although TGCT predominantly affects skeletally mature adults, TGCT has also been reported in patients as young as 8-months-old [5]. TGCT in pediatrics is exceptionally rare with an incidence rate of 2.86 cases per million people for L-TGCT (excluding digits) and 1.30 cases per million for D-TGCT reported in the Netherlands, with the knee as the most common location (46% for L-TGCT and 66% for D-TGCT) [6].

Adults with TGCT experience a wide range of clinical presentations, from asymptomatic to debilitating, often facing a significant disease burden. Quality of life is impacted not only by the disease itself but also by repeat surgical interventions, and treatment-related sequela [4, 7]. Few studies thus far have described pediatric TGCT which is believed to mimic the adult clinical presentation and disease course and none have captured the patient experience [6, 8–12].

It is speculated that TGCT in adults and pediatrics have the same molecular etiology characterized by a chromosomal aberration that disrupts the 3’ end of *colony stimulating factor 1 (CSF1)* gene [13–15]. Surgical management is the mainstay treatment for symptomatic patients with TGCT and systemic targeted therapies that inhibit the CSF1/CSF1R axis have also been approved in the United States, European Union, and China for symptomatic adults with TGCT [16–19]. However, drug development, thus far, has focused on the adult patient population, leaving a critical unmet need for advancements in pediatric TGCT populations.

Real-world data are needed to characterize the impact of TGCT on pediatric patients’ quality of life and to understand similarities and differences between pediatric and adult patients. The primary objective of this study is to describe the natural history and patient experience of TGCT in pediatric patients and to compare disease impact across pediatric and adult populations using the largest global patient registry from TGCT Support.

## Methods

### Survey population

TGCT Support, a program of the Life Raft Group, is an international, non-profit patient advocacy organization that initiated a longitudinal, multinational observation registry to capture real-world data on patient-reported experience and describe the typical journey, burden of disease, and unmet need. The TGCT Support Registry includes a questionnaire that collects baseline characteristics and changes made in respondents’ experiences every 6 months. The questionnaire allows patients (when appropriate), caregiver, or legally authorized representatives to fill out the survey. Patients learn about the registry through TGCT Support newsletters, webinars, and patient support groups. To minimize selection bias, no individual outreach or targeted recruitment is conducted. Internal validity is assessed through multiple registry questions that cross-check responses, such as number of surgeries, types of procedures, and corresponding dates.

The current cross-sectional analysis includes the initial entry of a subject as determined by entry submission between October 06, 2022, to November 26, 2024. At the data cutoff, 163 pediatric patients with TGCT were recorded (Supplementary Fig. 1). For the analyses, eligible patients were < 18 years of age at the time of last response, with a diagnosis of TGCT, and a complete entry. A total of 122 pediatric patients met eligibility criteria. Given the rarity of pediatric TGCT and descriptive nature of this study, all eligible patients who were able to participate during the study period were included, maximizing the sample size achievable. As such, this study does not have a pre-specified sample size calculation, and the findings should be interpreted in the context of the available population.

The institutional review board, Advarra (Columbia, Maryland), provided approval for the analysis of the TGCT Support Registry, and written informed consent was obtained from each patient (when appropriate), caregiver or legally authorized representative(s) who participated in the registry.

### Survey design

An electronic questionnaire was developed using RedCap, an electronic data capture tool. The registry consists of 177 fields with branching logic to record clinical information, and patient-centered QoL survey questions. The questionnaire captures information regarding patient demographics, patient reported disease and clinical characteristics, diagnostic experiences (e.g., time to diagnosis, misdiagnoses), treatments received (e.g., surgery, surgical techniques, medications, radiotherapy), symptom management, follow-up care and imaging frequency, quality of life (e.g., function, pain, and activities of daily living), and patient journey as previously described [4]. This cross-sectional analysis is compared to Stern et al. [4] to determine differences among pediatric and adults. Disease subtype was self-reported as diffuse, localized, or unknown.

### Statistical analysis and reporting

All analyses were performed in SAS v9.4. Descriptive statistics were generated and stratified across TGCT subtypes: diffuse, localized, or unknown.

This study was exploratory and hypothesis-generating; therefore, formal hypothesis testing was not the primary aim. To identify potential relationships for future investigation, comparisons between D-TGCT and L-TGCT were performed separately in pediatric and adult populations using χ² tests for categorical variables and two-tailed t-tests for continuous variables, with an unadjusted p value < 0.05 considered statistically significant. Similarly, comparison between pediatric and adult TGCT were performed separately using the same statistical tests. All estimates were unadjusted for potential confounding variables, and p values were not reported when the frequency of treatment events in any cell was fewer than five.

Additional analyses were conducted to explore the potential impact of confounding variables on misdiagnosis and surgical burden. Conditional logistic regression was used to evaluate the association between TGCT subtype and misdiagnosis in pediatrics, adjusting for age at diagnosis, sex, disease location, and diagnosing healthcare provider (HCP). An additional conditional logistic regression was used to evaluate factors associated with misdiagnosis between pediatric patients and adults adjusted for TGCT subtype, sex, disease location, and diagnosing HCP. A Poisson regression model was used to estimate risk ratios for the number of surgeries by TGCT subtype in pediatrics, adjusting for current age, age at diagnosis, sex, disease location, and diagnosing HCP.

This cross-sectional analysis is reported in accordance with the STROBE checklist.

## Results

### Baseline characteristics

Demographics are summarized for 122 pediatric patients with TGCT (Table 1). Pediatric patients were mostly female (*n* = 82, 67.2%), with a median age at diagnosis of 14.5 years (range: 3–17), and a median age at enrollment of 16 (range: 4–17). 90% of pediatric patients were considered adolescents at diagnosis, defined as > 11 years of age [20]. Nearly half of pediatric patients resided outside the United States (U.S.) and the most common country outside the U.S. includes Canada, United Kingdom, Italy, Germany, and Australia. Of the 122 pediatric patients, majority were diagnosed with D-TGCT (*n* = 89, 73.0%) and the knee was the most common location regardless of TGCT subtype (*n* = 102, 83.6%). Other joints involved include the hip (*n* = 13, 10.7%), the ankle (*n* = 5, 4.1%), and wrist or shoulder (*n* = 2, 1.6%).

**Table 1:** Pediatric patient characteristics

	Diffuse (*n* = 89, 73.0%)	Localized (*n* = 20, 16.4%)	Unknown (*n* = 13, 10.6%)	Total (*N* = 122)
**Female,** ***n*** **(%)**	59 (66.3)	14 (70.0)	9 (69.2)	82 (67.2)
**Median age at Diagnosis**,** years (range)**	14 (3–17)	15 (4–17)	14.5 (7–15)	14.5 (3–17)
**Median age at enrollment**,** years (range)**	16 (4–18)	17 (6–18)	15 (10–18)	16 (4–18)
**Located in the US**,** n (%)**	47 (52.8)	11 (55.0)	4 (30.8)	62 (50.8)
**Age Group**,** n (%)**				
Children (2–11 years)	8 (9.0)	3 (15.0)	1 (7.7)	12 (9.8)
Adolescents (12–18)	81 (91.0)	17 (85.0)	12 (92.3)	110 (90.2)
**Race**,** n (%)**				
White	79 (88.8)	19 (95.0)	9 (69.2)	101 (87.7)
African American	1 (1.1)	0 (0.0)	0 (0.0)	1 (0.8)
Asian	6 (6.7)	0 (0.0)	1 (7.7)	7 (5.7)
Prefer not to say	1 (1.1)	0 (0.0)	0 (0.0)	1 (0.8)
Other	2 (2.3)	1 (5.0)	3 (23.1)	6 (4.9)
**Hispanic**,** n (%)**	10 (11.2)	3 (15.0)	2 (15.4)	15 (12.3)
**Location of disease**,** n (%)**				
Knee	76 (85.4)	16 (80.0)	10 (76.9)	102 (83.6)
Hip	8 (9.0)	4 (20.0)	1 (7.7)	13 (10.7)
Ankle	5 (5.6)	0 (0.0)	0 (0.0)	5 (4.1)
Other	0 (0.0)	0 (0.0)	2 (15.4)	2 (1.6)

### Diagnostic journey

Of the 122 pediatric patients, 62.3% had been misdiagnosed prior to receiving their TGCT diagnosis (*n* = 76) (Table 2). A conditional logistic regression model indicated that, after adjustments for age at diagnosis, sex, TGCT location, and diagnosing HCP, pediatric patients with D-TGCT had higher odds of receiving a misdiagnosis compared to those with L-TGCT (odds ratio [OR]: 1.8, 95% confidence interval [CI] 1.3, 2.2, *p* = 0.02) (Data Supplement, Table S1). Overall, differential diagnoses included generalized anxiety disorder, growing related pains, baker’s cysts, labrum or meniscus tears, juvenile rheumatoid arthritis, idiopathic rheumatoid arthritis, septic arthritis, sports injuries, ganglion cysts, ehlers danlos syndrome, algoneurodystrophy, tendonitis, and congenital anatomical defects. Baker’s cysts (*n* = 15, 19.7%), ligament and tendon tears, sprains and other sports injuries (*n* = 25, 32.9%), and juvenile rheumatoid arthritis (*n* = 11, 14.5%) were the most common misdiagnoses.

**Table 2:** Diagnostic journey

	Diffuse (*n* = 89, 73.0%)	Localized (*n* = 20, 16.4%)	Unknown (*n* = 13, 10.6%)	Total (*N* = 122)
**Misdiagnosis**	56 (62.9)	10 (50.0)	10 (76.9)	76 (62.3)
**Main Symptom that Led to Diagnosis**,** n (%)**				
Pain	37 (42.1)	12 (60.0)	6 (46.2)	55 (45.5)
Swelling	44 (49.4)	5 (25.0)	5 (38.5)	54 (44.3)
Stiffness	2 (2.3)	1 (5.0)	0 (0.0)	3 (2.5)
Limitations in Range of Motion	4 (4.6)	0 (0.0)	2 (15.4)	6 (5.0)
Other^**^	2 (2.3)	2 (10.0)	0 (0.0)	4 (3.3)
**Pediatric TGCT-Diagnosing HCP**,** n (%)**				
Orthopedic/sports medicine	62 (69.7)	12 (60.0)	5 (38.5)	79 (64.8)
Orthopedic oncologist	19 (21.4)	7 (35.0)	0 (0.0)	26 (21.3)
Medical oncologist	0 (0.0)	0 (0.0)	0 (0.0)	0 (0.0)
Rheumatologist	4 (4.5)	1 (5.0)	4 (30.8)	9 (7.4)
General Practitioner/Pediatrician	4 (4.5)	0 (0.0)	4 (30.8)	8 (6.6)
**Time from Symptom Onset to Diagnosis**,** n (%)**				
< 12 months	42 (47.1)	8 (40.0)	4 (30.8)	54 (44.3)
12–24 months	24 (27.0)	5 (25.0)	3 (23.1)	32 (26.2)
25–60 months	14 (15.7)	4 (20.0)	3 (23.1)	21 (17.2)
> 60 months	6 (6.7)	3 (15.0)	2 (15.4)	11 (9.0)
Diagnosed during Surgery	3 (3.4)	0 (0.0)	1 (7.7)	4 (3.3)

HCP, healthcare provider;

**’Other’ includes popping, cracking, and a palpable lump

Pain (*n* = 55, 45.5%) and swelling (*n* = 54, 44.3%) were the most common symptom that led patients to seek diagnosis (Table 2). Swelling was more common among pediatric patients with D-TGCT compared with L-TGCT (D-TGCT; *n* = 44, 49.4% vs. L-TGCT; *n* = 5, 25.0%). More than half (*n* = 64, 53%) of pediatric patients had symptoms for 12 months or longer before diagnosis. The HCP responsible for diagnosing TGCT was predominantly general orthopedists or orthopedics in sports medicine (*n* = 79, 64.8%), except for those with unknown subtype who were also diagnosed by rheumatologists (*n* = 4, 30.8%) or pediatricians (*n* = 4, 30.8%) (Table 2).

Majority of pediatric patients have had an intermittent increase in TGCT-related symptoms, commonly referred to as a ‘flare’, within the last 6 months (*n* = 82, 67.2%) (Data Supplement, Table S2). Pediatric patients with D-TGCT reported a flare significantly more often than pediatric patients with L-TGCT (*n* = 59, 66.3% vs. *n* = 9, 40.0%, *p* < 0.01). Non-steroidal anti-inflammatory drugs (*n* = 76, 62.3%) and over-the-counter analgesics (*n* = 93, 76.2%) were often used (Data Supplement, Table S2).

### Treatment

Tumor resection is the most common treatment strategy discussed at initial diagnosis (*n* = 115, 94.3%) (Data Supplement, Table S3). Systemic therapies (*n* = 19, 15.6%) and radiotherapy (*n* = 25, 20.5%) were occasionally discussed. Active surveillance was mentioned as an approach for 1 in 5 pediatric patients at initial diagnosis (*n* = 23, 18.9%). Upon diagnosis, pediatric patients (*n* = 75, 61.5%) are often treated within 3 months and orthopedic surgeons were the most common HCP involved in treatment (*n* = 98, 80.3%), followed by physiotherapists (*n* = 49, 40.2%), and orthopedic oncologists (*n* = 45, 36.9%) (Table 3). Medical oncologists were involved in one third of pediatric patients’ care (*n* = 41, 33.6%), namely those with D-TGCT (*n* = 37, 41.6%).

**Table 3:** From diagnosis to treating providers

	Diffuse (*n* = 89, 73.0%)	Localized (*n* = 20, 16.4%)	Unknown (*n* = 13, 10.6%)	Total (*N* = 122)
**Time from Diagnosis to Treatment,** ***n*** **(%)**				
< 1 Month	32 (36.0)	8 (40.0)	5 (38.5)	45 (36.9)
1–3 Months	22 (24.7)	7 (35.0)	1 (7.7)	30 (24.6)
4–6 Months	13 (14.6)	1 (5.0)	1 (7.7)	15 (12.3)
7–11 Months	8 (9.0)	2 (10.0)	0 (0.0)	10 (8.2)
≥ 12 Months	4 (4.5)	0 (0.0)	2 (15.4)	6 (4.9)
Active Surveillance	10 (11.2)	2 (10.0)	4 (30.8)	16 (13.1)
**Pediatric TGCT-Treating HCPs**,** n (%)**				
Orthopedic/sports medicine	74 (83.2)	16 (80.0)	8 (61.5)	98 (80.3)
Orthopedic oncologist	33 (37.1)	8 (40.0)	4 (30.8)	45 (36.9)
Medical oncologist	37 (41.6)	1 (5.0)	3 (23.1)	41 (33.6)
Rheumatologist	12 (13.5)	2 (10.0)	2 (15.4)	16 (13.1)
Physiotherapist	41 (46.1)	7 (35.0)	1 (7.7)	49 (40.2)
General Practitioner/Pediatrician	29 (32.6)	7 (35.0)	7 (53.9)	43 (35.3)

HCP, healthcare provider;

#### Surgery

Surgery was the mainstay treatment among all pediatric patients (*n* = 100, 82.0%) (Table 4). On average, pediatric patients with D-TGCT experienced more surgeries than pediatric patients with L-TGCT (3.4 surgeries ± 2.8 standard deviation [SD] vs. 1.8 ± 1.5 SD). After adjustment for location of disease, sex, age at entry, age at diagnosis, and HCP responsible for diagnosis in a Poisson loglinear analysis, D-TGCT remained significantly associated with the number of surgeries compared to L-TGCT (adjusted IRR: 1.8, 95% CI: 1.1, 2.9, *p* = 0.01) (Supplement Data, Table S4). No other predictors showed significant associations.

**Table 4:** Treatment modalities

	Diffuse (*n* = 89, 73.0%)	Localized (*n* = 20, 16.4%)	Unknown (*n* = 13, 10.6%)	Total (*N* = 122)	*p*-value
Tumor Resection, *n* (%)	76 (85.4)	16 (80.0)	8 (61.5)	100 (82.0)	0.55
Arthroscopic Surgery Open Synovectomy Combined Anterior/Posterior Synovectomy	56 (62.9) 44 (49.4) 26 (29.2)	8 (40.0) 8 (40.0) 3 (15.0)	6 (46.2) 2 (15.4) 0 (0.0)	70 (57.4) 54 (44.3) 29 (23.8)	0.11 0.45 -
Systemic Therapy, *Check all that apply* Pexidartinib Imatinib Nilotinib	20 (22.5) 7 (7.9) 12 (13.5) 1 (1.1)	0 (0.0) 0 (0.0) 0 (0.0) 0 (0.0)	1 (7.7) 1 (7.7) 0 (0.0) 0 (0.0)	21 (17.2) 8 (7.4) 12 (9.8) 1 (0.8)	-
Radiation External Beam Intra-articular Yttrium-90	0 (0.0) 0 (0.0) 0 (0.0)	0 (0.0) 0 (0.0) 0 (0.0)	0 (0.0) 0 (0.0) 0 (0.0)	0 (0.0) 0 (0.0) 0 (0.0)	-
Active Surveillance	43 (48.3)	9 (45.0)	8 (61.5)	60 (49.2)	0.79
**Average surgeries**,** (SD)** Median	3.4 (2.8) 2	1.8 (1.5) 1	1.1 (1.2) 1	2.8 (2.5) 2	0.01*
**Local Recurrences**,** n (%)**					
Yes 1 Recurrence ≥ 2 Recurrences	59 (66.3) 19 (21.3) 40 (44.9)	3 (15.0) 2 (10.0) 1 (5.0)	4 (3.8) 1 (7.7) 3 (23.1)	66 (54.1) 22 (18.0) 44 (36.1)	0.01*
No I have not had surgery I am unsure	30 (33.7) 12 (13.5) 8 (9.0)	17 (85.0) 2 (10.0) 4 (20.0)	9 (69.2) 5 (38.5) 1 (7.7)	56 (45.9) 19 (15.6) 13 (10.7)	

*denotes statistical significance between D-TGCT and L-TGCT determined by χ2 test or 2-sided t-test

Most pediatric patients have had ≥ 1 tumor resection (*n* = 100, 82.0%), including the following surgical approaches: arthroscopy (*n* = 70, 57.4%), open resection (*n* = 54, 44.3%), and combined anterior and posterior approach for TGCT of the knee (*n* = 29, 23.8%). Arthroscopies were nominally more common in pediatric patients with D-TGCT compared to L-TGCT (*n* = 56, 62.9% vs. *n* = 8, 40.0%, *p* = 0.11). Open approaches and combined approaches were reported in similar proportions in patients with D-TGCT and L-TGCT.

The local recurrence rate (LRR) was 54.1% (*n* = 66) in the total population and 66.0% (*n* = 66) in the population of pediatric patients with prior resections (*n* = 100). LRR was higher among pediatric patients with D-TGCT compared to pediatric patients with L-TGCT (*n* = 59, 66.3% vs. *n* = 3, 15.0%, *p* = 0.01). Subsequent recurrences following additional resections were high among pediatric patients with D-TGCT (*n* = 40, 67.8%).

#### Systemic therapies

Systemic therapies were occasionally prescribed (*n* = 21, 17.2%)(Table 4). All systemic therapies were prescribed off-label, as there are no approved indications for pediatric patients with TGCT. Systemic therapies were exclusively used in pediatric patients with D-TGCT and in one patient with an unknown subtype of TGCT. Eighty-one percent of pediatric patients who received systemic therapies (*n* = 17) have received one line of treatment, while four patients received two lines of treatment. Imatinib (*n* = 12, 57.1%) was the most commonly used systemic therapy, followed by pexidartinib (*n* = 8, 38.1%)(Table 4). Three patients received imatinib followed by pexidartinib and one patient with D-TGCT received imatinib followed by nilotinib. Systemic therapy dosage aligned with adult indications (e.g., 500 mg total for pexidartinib and 400 mg for imatinib).

Pediatric patients or their caregivers were asked about their concerns regarding systemic therapies (*n* = 21). The most reported concerns included fertility, lifestyle changes associated with aging (such as transitioning from high school to college), and the lack of data to support use in pediatric populations.

### Burden of disease

Pain was the most common symptom (*n* = 115, 94.0%), regardless of TGCT subtype (D-TGCT: *n* = 86, 96.6% vs. L-TGCT: *n* = 18, 90.0%) (Data Supplement, Table S5). Eighty-nine percent of patients with TGCT reported limited range of motion (*n* = 109), and patients with D-TGCT or L-TGCT experienced similar limitations in range of motion (*n* = 81, 91.0% vs. *n* = 17, 85.0%). Pediatric patients with D-TGCT experienced swelling more frequently than those with L-TGCT (*n* = 79, 88.7% vs. *n* = 12, 60.0%, *p* < 0.01). In the 7-day period prior to their entry, pain interfered in some degree with most pediatric patients’ enjoyment of life (*n* = 98, 80.3%) (Data Supplement, Table S6). Similarly, pain interfered with pediatric patients’ enjoyment of fun activities (*n* = 101, 82.8%).

Patients reported that TGCT impacted their ability to participate in sports, future career planning, and school choices. One patient recalls that they intended to join the military like their parent, however, they stated that TGCT will be the reason they can’t pass the physical exams. Several pediatric patients mentioned TGCT impacting their ability to focus on school or relate to their friends.

### Pediatric TGCT compared to adult TGCT

Overall, TGCT impacts adult and pediatric patients with similar burden and outcomes; however, several journey characteristics differed (Table 5). Comparably, adult and pediatric patients reported pain (adult; *n* = 210, 42.3% vs. pediatric; *n* = 55, 45.5%) and swelling (adult: *n* = 189, 38.0% vs. pediatric: *n* = 54, 43.0%) as the most common symptom that led to a sought-after diagnosis and pain was the most common symptom throughout their journey (*n* = 457, 92.0% vs. *n* = 115, 94.3%). No differences between pediatric and adult patients were noted based on subtype of TGCT.

**Table 5:** Comparison between pediatric and adult TGCT patient journey

Variables	Pediatric TGCT (n=122)			Adult TGCT (n=497)			Pediatric vs Adult		
	Localized (*n* = 20)	Diffuse (*n* = 89)	Unknown (*n* = 13)	Localized (*n* = 94)	Diffuse (*n* = 355)	Unknown (*n* = 48)	Localized: *p* value	Diffuse: *p* value	Unknown: *p* value
**Female, n (%)**	14 (70.0)	59 (66.3)	9 (69.2)	76 (80.9)	254 (71.5)	40 (83.3)	0.17	0.55	0.09
**US, n (%)**	11 (55.0)	47 (52.8)	4 (30.8)	53 (56.4)	203 (57.2)	24 (50.0)	0.90	0.43	0.19
**TGCT of the knee, n (%)**	16 (80.0)	76 (85.4)	10 (76.9)	57 (72.2)	256 (72.2)	26 (54.2)	0.56	0.33	0.14
**Misdiagnosis, n (%)**	10 (50.0)	56 (62.9)	10 (76.9)	41 (51.3)	182 (51.3)	25 (52.1)	0.78	0.35	0.003
**Main Symptom that led to diagnosis, n (%)**									
Pain, n (%)	12 (60.0)	37 (42.1)	6 (46.2)	48 (51.0)	141 (39.7)	21 (43.7)	0.47	0.62	0.88
Swelling, n (%)	5 (25.0)	44 (49.4)	5 (38.5)	23 (24.5)	152 (42.8)	14 (29.2)	0.96	0.39	0.52
**Diagnosing HCPs, n (%)**									
Orthopedic/Sports Medicine	12 (60.0)	62 (69.7)	5 (38.5)	51 (54.3)	222 (62.5)	31 (64.5)	0.64	0.44	0.09
Orthopedic Oncologist	7 (35.0)	19 (21.4)	0 (0.0)	30 (31.9)	83 (23.4)	5 (10.4)	0.79	0.65	0.22
GP/PCP/Pediatrician	0 (0.0)	4 (4.5)	4 (30.8)	3 (3.2)	12 (3.4)	3 (6.3)	0.42	0.89	0.08
**Duration from Symptom Onset to Diagnosis, n (%)**									
< 12 months	8 (40.0)	42 (47.1)	4 (30.8)	39 (41.5)	132 (37.2)	14 (29.2)	0.89	0.13	0.91
≥ 12 months	12 (60.0)	44 (49.4)	8 (61.5)	50 (53.2)	201 (56.6)	27 (56.3)			
**Duration from Diagnosis to Treatment, n (%)**									
< 12 months	18 (90.0)	75 (84.2)	7 (53.8)	69 (73.4)	240 (67.6)	21 (43.8)	0.34	0.14	0.23
≥ 12 months	0 (0.0)	4 (4.5)	2 (15.4)	4 (4.3)	35 (9.8)	4 (8.3)			
**Treating HCP, n (%)**									
Orthopedics/Sports Medicine	16 (80.0)	74 (83.2)	8 (61.5)	75 (79.8)	264 (74.4)	32 (66.7)	0.98	0.08	0.73
Orthopedic Oncologist	8 (40.0)	33 (37.1)	4 (30.8)	53 (56.4)	238 (67.0)	15 (31.3)	0.18	0.65	0.97
Medical Oncologist	1 (5.0)	37 (41.6)	3 (23.1)	6 (6.4)	122 (34.4)	11 (22.9)	0.82	0.20	0.99
**Treatments, n (%)**									
Arthroscopy	8 (40.0)	56 (62.9)	6 (46.2)	32 (34.0)	167 (47.0)	18 (37.5)	0.81	0.04	0.57
Open surgery	8 (40.0)	44 (49.4)	2 (15.4)	42 (44.7)	168 (47.3)	8 (16.7)	0.77	0.59	0.91
Systemic Therapy	0 (0.0)	20 (22.5)	1 (7.7)	3 (3.2)	86 (24.2)	6 (12.5)	0.42	0.74	0.63
Active Surveillance	9 (45.0)	43 (48.3)	8 (61.5)	30 (31.9)	153 (43.1)	18 (37.5)	0.26	0.36	0.12
average surgeries ± SD	1.8 ± 1.5	3.4 ± 2.8	2.9 ± 2.5	1.8 ± 1.6	2.8 ± 2.7	1.7 ± 1.1	0.95	0.39	0.18
**LRR after surgery**	n=17	n=76	n=8	n=68	n=289	n=36			
1 or more Recurrence(s), n (%)	3 (17.6)	59 (77.6)	4 (50.0)	23 (33.8)	207 (71.6)	16 (44.4)	0.13	0.76	0.76

HCP, healthcare provider; GP, general practitioner; LRR, local recurrence rate; PCP, primary care provider

*p value determined by Mantel-Haenszel χ2 test or t-test by TGCT subtype

However, pediatric patients with TGCT experienced misdiagnoses significantly more frequently than adults (*n* = 76, 62.3% vs. *n* = 248, 49.9%, p *<* 0.01). Consistent with these findings, a conditional logistic regression model adjusted for sex, TGCT subtype, and diagnosing HCP, indicated that pediatric patients had significantly higher odds of receiving a misdiagnosis compared with adults (OR 1.2; 95% CI, 1.1, 1.5; *p* = 0.02) (Supplementary Table S7). When stratified by subtype, pediatric patients with TGCT of unknown subtype were misdiagnosed more often than adults with unknown TGCT (*n* = 10, 76.9% vs. *n* = 25, 52.1%, *p* < 0.01), whereas misdiagnosis in pediatric patients compared to adults with L-TGCT did not differ (*n* = 10, 50% vs. *n* = 41, 51.3%, *p* = 0.78). Misdiagnoses were also numerically higher among pediatric patients with D-TGCT than adults, although this difference did not reach statistical significance (*n* = 56, 62.9% vs. *n* = 182, 51.3%, *p* = 0.35). Among pediatric patients that were misdiagnosed, psychological conditions such as generalized anxiety disorder was reported as alternative diagnoses. Such psychological diagnoses were not reported as differential diagnoses or misdiagnoses among adults with TGCT.

Despite the high proportion of misdiagnoses among pediatric patients, the duration from symptom onset to diagnosis among adult and pediatric patients was consistent, regardless of TGCT subtype. Additionally, majority of symptoms were managed similarly among adults and pediatric patients. Although, pediatric patients received joint aspirations significantly more frequently than adult patients (*n* = 58, 47.5% vs. *n* = 112, 22.5%, *p* < 0.05), likely representing the lack of oncology input in the management of TGCT.

Treatment approaches discussed were similar among pediatric and adult patients and most patients were treated within 3 months of their TGCT diagnosis, highlighting that patients are treated quickly once diagnosed. Although both pediatric patients and adults were diagnosed frequently by general orthopedic surgeons or sports medicine surgeons, adult patients with TGCT involved orthopedic oncologists in their care significantly more often than pediatric patients with TGCT (*n* = 306, 61.6% vs. *n* = 45, 36.9%, *p* < 0.01). The management of TGCT in pediatrics commonly involved primary care (i.e. pediatricians).

Surgery was the primary treatment for both adult and pediatric patients with TGCT. Arthroscopies were more commonly performed on pediatric patients with TGCT than adult patients (*n* = 70, 57.4% vs. *n* = 217, 43.7%) and that difference is largely driven by pediatrics with D-TGCT (*n* = 56, 62.9% vs. *n* = 176, 47.0%, *p* = 0.04) (Table 5). The mean number of surgical resections was similar among adult and pediatric patients (2.7 vs. 2.8), as were the LRR following surgery (*n* = 246, 62.6% vs. *n* = 66, 66.0%).

Despite there being no approved systemic therapies available for patients under 18 years of age, medical oncologists were involved in pediatric patients’ care in a similar proportion as adult patients (*n* = 41, 33.6% vs. *n* = 139, 28.0% respectively). The use of systemic therapies was similar among pediatric and adult patients (*n* = 21 17.2% vs. *n* = 95, 19.1%) (Table 5). Among patients on systemic therapies, pediatric patients received imatinib more frequently than adults (*n* = 12, 57.1% vs. *n* = 37, 39.0%), whereas pexidartinib was the most common systemic therapy among adults (*n* = 21, 38.1% vs. *n* = 56, 59.0%).

## Discussion

**Fig. 1:**
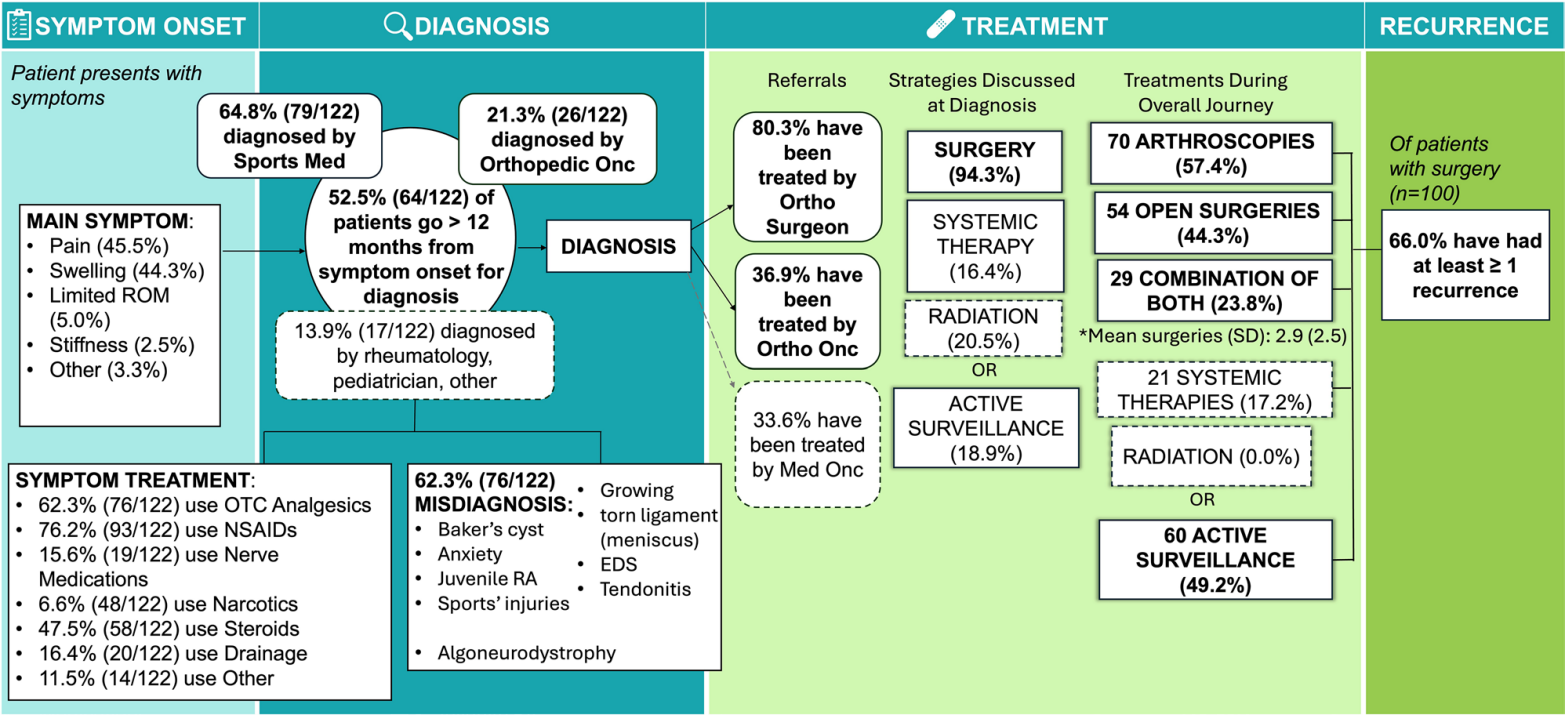
Pediatric patient journey from diagnosis to treatment. EDS, elder’s danlos syndrome; NSAID, nonsteroidal anti-inflammatory drugs; OTC, over the counter; RA, rheumatoid arthritis

This registry is the largest and most comprehensive dataset of pediatric TGCT to date, and the first to capture disease characteristics, treatments, and outcomes from a patient perspective. Previously, the largest pediatric study included 29 children (ages 3–11) over a 16-year period and focused on imaging and 1–2 year recurrence rates [12], while a 2018 meta-analysis reviewed 76 patients from 17 case reports, noting similarities to adult disease [6]. Our findings demonstrate that, like adults, pediatric patients with TGCT face a long diagnostic journey, unmet treatment needs, and significant disease burden (Fig. 1). Pediatric patients also had high misdiagnosis rates (62.3%), less frequent orthopedic oncologic care compared to adults (36.9% vs. 61.6% for adults), and often had incomplete subtype classification when diagnosed by rheumatologists or pediatricians, suggesting limited familiarity with newer TGCT nomenclature. Despite higher misdiagnosis rates, time from symptom onset to diagnosis was similar for pediatric and adult patients, who also reported similar symptoms, most commonly pain and swelling. Pediatric patients with L-TGCT and D-TGCT reported similarly impaired range of motion, which may reflect localized tumors obstructing movement, such as in the patellofemoral joint. However, the interpretation of these findings is limited by the self-reported nature of data, preventing objective clinical or anatomical assessment. The rate of misdiagnosis and duration between accurate diagnosis and surgery may be influenced by the disease location. Although in a conditional logistic regression analysis evaluating misdiagnosis in pediatric patients with D-TGCT and L-TGCT, adjusted for disease location and other factors, we found that location alone did not fully explain the relationship between misdiagnosis and TGCT subtype. However, TGCT of unknown subtype was not evaluated in this model. Misdiagnoses were more often reported among pediatric patients with unknown subtype, and several patients presented with TGCT at less common anatomical sites. These findings suggest that TGCT location may contribute to diagnostic uncertainty and subtype characterization, highlighting the importance of considering TGCT in the differential diagnosis of lesions at atypical anatomical sites [21–23].

Surgery is the mainstay treatment for both pediatric and adult patients with TGCT, as expected. Pediatric TGCT mimicked the chronic nature of the disease experienced by adults including the high LRR. Previous research has demonstrated no difference in the 2.5-year LRR after surgical treatment in pediatric patients compared to adults (15% vs. 11% in L-TGCT; 47% vs. 44% in D-TGCT) [6], which is consistent with our findings. However, our analysis demonstrate higher overall LRR and subtype-associated LRR as compared with those reported in the Dutch study for both adults and pediatric (Table 5) [6]. This may reflect a selection bias of patients enrolled in our registry or a severity bias where patients in our study, often treated outside specialty centers, have higher LRR and multiple surgeries. Most studies in TGCT occur at academic specialty centers and our results may reflect real-world community settings, independent of institution and geography, leading to substantial variability in care and expertise and wide confidence intervals [4]. Additionally, in our study, post-operative residual disease is not ruled out and LRR may be over-reported due to inconsistent post-operative follow-up imaging. Notably, LRR remained high (67.8%) following subsequent surgical resections of D-TGCT, consistent with the 75% LRR reported among adults with D-TGCT and prior surgeries outside tertiary specialty centers described by Mastboom and colleagues [24].

Although radiation was discussed as a treatment for pediatric TGCT, no pediatric patient received radiation therapy to treat TGCT. This contrasts with adults with TGCT where 7.8% of patients received radiation [4]. It is possible that pediatric patients were less likely to receive radiotherapy due to the growing concerns for long-term risks including fibrosis and joint stiffness, radiation-induced malignancies, malignant transformation, as well as a lack of efficacy established for disease control in a younger population, and the increasing availability of systemic therapies. Importantly, a global position paper on this topic underscore that the available literature provides insufficient data to propose reliable recommendations for the use of radiotherapy as a standard treatment for TGCT [2].

Systemic therapies were used in similar proportions in pediatric and adult TGCT, despite approved indications being limited to adults. Pediatric use of systemic therapies was off-label, with imatinib most common. Willingness to use systemic therapy was noted, though concerns were raised regarding fertility impact, lifestyle changes, and lack of pediatric safety and efficacy data. Preliminary evidence from population pharmacokinetic analysis suggests that similar exposures were achieved by adult and pediatric patients following pexidartinib 800 mg [25]. However, insufficient evidence is available to determine whether the same dose is effective in the TGCT pediatric population or whether lower doses could be effective. In this regard, only two of the 29 clinical studies listed on ClinicalTrials.gov included pediatric patients. In particular, none of the 3 pivotal trials with pexidartinib (*n* = 61), vimseltinib (*n* = 83), and pimicotinib (*n* = 63) in TGCT enrolled pediatric patients, and results await whether the phase 3 TANGENT trial for emactuzumab included patients > 12 years (https://clinicaltrials.gov/ NCT#05417789). The second study was an investigator-initiated trial specifically designed to evaluate safety, tolerability, and efficacy in pediatric patients (https://clinicaltrials.gov/ NCT#02390752). Drug development programs should consider enrolling pediatric patients in clinical trials to determine if the dose used for adults is sufficient for various age groups given the similar disease burden, high LRR, unmet need, and clinical characteristics.

Our cross-sectional analysis is the first to describe the journey of pediatric patients with TGCT. On the other hand, a limitation of our work is that multiple comparisons were performed without formal adjustment for multiplicity. These analyses did not adjust for potential confounders due to the descriptive intent of the study. Therefore, observed associations might reflect underlying differences between patients rather than true age effect and should be considered hypothesis-generating until confirmed in prospective studies that control for potential confounding variables such as healthcare access and region. Like many cross-sectional, self-reported registries, our study is subject to common limitations that are inherent to retrospective observational studies such as selection and recall bias. Registry participants may not represent the broader patient population. Patients may misremember or inaccurately report medical events such as perceiving differential diagnoses as misdiagnoses or reporting radiologic re-appearance, post-surgical residual disease, or symptomatic relapse as recurrence, potentially inflating reported LRR. Despite these limitations, patient registries offer valuable insights into patient experiences, independent of institution and geography, but these findings should be interpreted in light of these limitations.

Overall, our analysis highlights that pediatric patients with TGCT have significant symptoms, namely nonspecific symptoms such as pain and monoarticular joint swelling, and disease burden similar to adult patients. The nonspecific symptoms, rarity of disease, and lack of awareness may lead to significant delays in diagnosis and a high misdiagnosis rate. Currently, treatment strategies for pediatric TGCT do not differ substantially from those used in adults; however, certain pediatric-specific considerations must be considered. We propose a treatment paradigm which takes into consideration the current practices (Supplementary Fig. 2). This analysis and paradigm highlight that pediatric patients benefit from multidisciplinary management of TGCT including pediatric oncologists, pediatric orthopedic surgeons, and other dedicated specialists. The expert community, together with patient advocates, should continue to raise awareness of TGCT among pediatricians and other general specialists to enhance appropriate referral of patients for TGCT management in specialized centers from the onset of the disease, thus, facilitating the development of tailored approaches for pediatric patients beyond repeat surgeries. Further research is warranted to elucidate the optimal treatment strategy in pediatrics. 

## Electronic Supplementary Material

Below is the link to the electronic supplementary material.

Supplementary Material 1

## Data Availability

The datasets generated and/or analyzed during the current study are not publicly available due to the way the patient consent form was written but aggregated data is available upon request to the corresponding author.

## Abbreviations

CSF1: Colony stimulating factor 1
D-TGCT: Diffuse tenosynovial giant cell tumor
LRR: Local recurrence rate
L-TGCT: Localized tenosynovial giant cell tumor
PVNS: Pigmented villonodular synovitis
TGCT: Tenosynovial giant cell tumor
