# Supplementary material for: Pediatric patients with tenosynovial giant cell tumor: real-world evidence from an observational registry
Source: Orphanet J Rare Dis. 2026 Jan 30;21:68. doi: 10.1186/s13023-026-04231-7 (PMC12922283; doi:10.1186/s13023-026-04231-7)
Supplement: Supplementary file 1 — Supplementary Material 1 [file 13023_2026_4231_MOESM1_ESM.docx]

Supplementary Tables

Table 1. Misdiagnosis by TGCT Subtype using Conditional Logistic Regression adjusted for sex, age at diagnosis, diagnosing provider, and disease location

|  | Misdiagnosis | No Misdiagnosis | Adjusted OR (95% CI) | *p-*value |
| --- | --- | --- | --- | --- |
| D-TGCT  L-TGCT (ref) | 56 (62.9)  10 (50.0) | 33 (37.1)  10 (50.0) | 1.8 (1.3, 2.2) | 0.02 |

CI, confidence interval; D-TGCT, diffuse tenosynovial giant cell tumor; OR, odds ratio; L-TGCT, localized tenosynovial giant cell tumor

Table 2. Symptom management and flares

|  | **Diffuse**  (n=89, 73.0%) | **Localized**  (n=20, 16.4%) | **Unknown**  (n=13, 10.6%) | **Total**  (N=122) |
| --- | --- | --- | --- | --- |
| **Flare in The Last 6 Months, n (%)** | 59 (66.3) | 9 (40.0) | 10 (76.9) | 82 (67.2) |
| **Supportive/Management of Symptoms, n (%)** | | | | |
| OTC Analgesics | 56 (62.9) | 12 (60.0) | 8 (61.5) | 76 (62.3) |
| NSAIDs | 66 (74.2) | 16 (80.0) | 11 (84.6) | 93 (76.2) |
| Nerve Medications (Gabapentin, Pregabalin) | 15 (16.9) | 1 (5.0) | 3 (23.1) | 19 (15.6) |
| Narcotics | 6 (6.7) | 0 (0.0) | 2 (15.4) | 8 (6.6) |
| Steroids | 40 (44.9) | 4 (20.0) | 4 (30.8) | 48 (39.3) |
| Drainage/Joint Aspirations | 49 (55.1) | 3 (15.0) | 6 (46.2) | 58 (47.5) |
| I did not use any treatment to help symptoms | 14 (15.7) | 3 (15.0) | 3 (23.1) | 20 (16.4) |
| Other | 10 (11.2) | 2 (10.0) | 2 (15.4) | 14 (11.5) |

OTC, over-the-counter; NSAIDs, nonsteroidal anti-inflammatory drugs

Table 3. Treatments discussed at initial diagnosis

|  | **Diffuse**  (n=89, 73.0%) | **Localized**  (n=20, 16.4%) | **Unknown**  (n=13, 10.6%) | **Total**  (N=122) |
| --- | --- | --- | --- | --- |
| **Treatments Discussed at Diagnosis, n (%)** *Check all that apply* | | | | |
| Surgery (arthroscopic or open) | 86 (96.6) | 20 (100.0) | 9 (69.2) | 115 (94.3) |
| Systemic Therapy  Pexidartinib  Imatinib/Nilotinib  Clinical Trials | 15 (16.9)  5 (5.6)  9 (10.1)  1 (1.1) | 2 (10.0)  1 (5.0)  1 (5.0)  0 (0.0) | 2 (15.4)  0 (0.0)  2 (15.4)  0 (0.0) | 19 (15.6)  6 (4.9)  12 (9.8)  1 (0.8) |
| Radiation | 20 (22.5) | 1 (5.0) | 4 (30.8) | 25 (20.5) |
| Active Surveillance | 15 (16.9) | 3 (15.0) | 5 (38.5) | 23 (18.9) |

Table 4. Adjusted Incidence Rate for Number of Surgeries by TGCT Subtype

| Variable | Level | β (Estimate) | Adjusted IRR (95% CI) | p value | Type 3 LR χ² | Type 3 LR p value |
| --- | --- | --- | --- | --- | --- | --- |
| **Subtype** | Diffuse Localized (Ref) Unknown | 0.58 0.0 -0.15 | 1.8 (1.1, 2.9) - 0.9 (0.35, 2.1) | 0.02 - 0.74 | 9.0 | 0.01 |
| **Location of TGCT** | - | -0.04 | 1.0 (0.8, 1.1) | 0.57 | 0.35 | 0.56 |
| **Sex** | - | 0.003 | 1.0 (0.7, 1.5) | 0.99 | 0.00 | 0.99 |
| **Age at Response** | - | -0.01 | 0.9 (0.89, 1.1) | 0.48 | 0.54 | 0.45 |
| **Age at Diagnosis** | - | -0.02 | 1.0 (0.93, 1.0) | 0.50 | 0.55 | 0.50 |
| **Diagnosing Provider** | - | -0.04 | 1.0 (0.8, 1.2) | 0.70 | 0.15 | 0.70 |

IRR, incidence risk ratio; LR, Likelihood Ratio

Table 5. Symptoms

|  | **Diffuse**  (n=89, 73.0%) | **Localized**  (n=20, 16.4%) | **Unknown**  (n=13, 10.6%) | **Total**  (N=122) |
| --- | --- | --- | --- | --- |
| **Symptoms Reported, n (%)** | | | | |
| Pain | 86 (96.6) | 18 (90.0) | 11 (84.6) | 115 (94.3) |
| Swelling | 79 (88.7) | 12 (60.0) | 12 (92.3) | 103 (84.4) |
| Limited Range of Motion | 81 (91.0) | 17 (85.0) | 11 (84.6) | 109 (89.3) |
| Stiffness | 75 (84.3) | 12 (60.0) | 10 (76.9) | 97 (79.5) |
| Instability | 60 (67.4) | 10 (50.0) | 7 (53.9) | 77 (63.1) |
| Giving Out/Away | 48 (53.9) | 8 (40.0) | 6 (46.2) | 62 (50.8) |
| Catching | 38 (42.7) | 4 (20.0) | 6 (46.2) | 48 (39.3) |
| Locking | 47 (52.8) | 7 (35.0) | 7 (53.9) | 61 (50.0) |
| Popping | 44 (49.4) | 10 (50.0) | 7 (53.9) | 61 (50.0) |
| Clicking | 48 (58.9) | 5 (25.0) | 5 (38.5) | 58 (47.5) |
| Grinding | 31 (34.8) | 6 (30.0) | 4 (30.8) | 41 (33.6) |
| Pressure | 45 (50.6) | 8 (40.0) | 2 (15.4) | 55 (45.1) |
| Weakened/tired/lack of muscle strength | 55 (61.8) | 10 (50.0) | 5 (38.5) | 70 (57.4) |
| Heat/Hot to Touch | 50 (56.2) | 4 (20.0) | 7 (53.9) | 61 (50.0) |
| Sensitivity/Discomfort | 56 (62.9) | 11 (55.0) | 7 (53.9) | 74 (60.7) |
| Enlargement | 30 (33.7) | 5 (25.0) | 5 (38.5) | 40 (32.8) |

Table 6. Burden of Disease

| **In the past 7 days…** | **Diffuse**  (n=89, 73.0%) | **Localized**  (n=20, 16.4%) | **Unknown**  (n=13, 10.6%) | **Total**  (N=122) |
| --- | --- | --- | --- | --- |
| **Pain Interfere in day-to-day activities, n (%)**  Not at all  To any degree | 12 (13.5)  77 (86.5) | 8 (40.0)  12 (60.0) | 3 (23.1)  10 (76.9) | 23 (18.9)  99 (81.1) |
| **Pain Interfere in social activities, n (%)**  Not at all  To any degree | 15 (16.9)  74 (83.1) | 7 (35.0)  13 (65.0) | 3 (23.1)  10 (76.9) | 25 (20.5)  97 (79.5) |
| **Pain Interfere in enjoyment of life, n (%)**  Not at all  To any degree | 16 (18.0)  73 (82.0) | 6 (30.0)  14 (70.0) | 2 (15.4)  11 (84.6) | 24 (19.7)  98 (80.3) |
| **Pain Interfere in enjoyment in fun activities, n (%)**  Not at all  To any degree | 13 (14.6)  76 (85.4) | 7 (35.0)  13 (65.0%) | 1 (7.7)  12 (92.3) | 21 (17.2)  101 (82.8) |

Table 7. Misdiagnosis by Pediatric and Adult Patients using Conditional Logistic Regression adjusted for sex, TGCT subtype, diagnosing provider, and disease location

|  | Misdiagnosis | No Misdiagnosis | Adjusted OR (95% CI) | *p-*value |
| --- | --- | --- | --- | --- |
| Adults (ref)  Pediatrics | 248 (49.9)  76 (62.3) | 249 (50.1)  46 (37.7) | 1.2 (1.1, 1.5) | 0.02 |

CI, confidence interval; D-TGCT, diffuse tenosynovial giant cell tumor; OR, odds ratio; L-TGCT, localized tenosynovial giant cell tumor

Supplementary Figure

Figure 1. Pediatric TGCT Registry Study Design. Data cut-off was October 06 2022 to November 26, 2024.

Figure 2. Proposed Treatment Paradigm for pediatric patients with TGCT
